# Does Checklist Implementation Improve Quantity of Data Transfer: An Observation in Postanesthesia Care Unit (PACU)

**DOI:** 10.4236/ojanes.2017.74007

**Published:** 2017-04

**Authors:** Lauren S. Park, Gloria Yang, Kay See Tan, Charlotte H. Wong, Sabine Oskar, Ruth A. Borchardt, Luis E. Tollinche

**Affiliations:** 1 The Warren Alpert Medical School of Brown University, Providence, RI, USA; 2 Department of Anesthesiology and Critical Care Medicine, Memorial Sloan Kettering Cancer Center, New York City, NY, USA; 3 Department of Epidemiology and Biostatistics, Memorial Sloan Kettering Cancer Center, New York City, NY, USA; 4 Cornell University, Ithaca, NY, USA

**Keywords:** Checklist, PACU, Recovery Room, Data Transfer, Handoff

## Abstract

**Background:**

Omission of patient information in perioperative communication is closely linked to adverse events. Use of checklists to standardize the handoff in the post anesthesia care unit (PACU) has been shown to effectively reduce medical errors.

**Objective:**

Our study investigates the use of a checklist to improve quantity of data transfer during handoffs in the PACU.

**Design:**

A cross-sectional observational study.

**Setting:**

PACU at Memorial Sloan Kettering Cancer Center (MSKCC); June 13, 2016 through July 15, 2016.

**Patients, other participants:**

We observed the handoff reports between the nurses, PACU midlevel providers, anesthesia staff, and surgical staff.

**Intervention:**

A physical checklist was provided to all anesthesia staff and recommended to adhere to the list at all observed PACU handoffs.

**Main outcome measure:**

Quantity of reported handoff items during 60 pre- and 60 post-implementation of a checklist.

**Results:**

Composite value from both surgical and anesthesia reports showed an increase in the mean report of 8.7 items from pre-implementation period to 10.9 post-implementation. Given that surgical staff reported the mean of 5.9 items pre-implementation and 5.5 items post-implementation without intervention, improvements in anesthesia staff report with intervention improved the overall handoff data transfer.

**Conclusions:**

Using a physical 12-item checklist for PACU handoff increased overall data transfer.

## 1. Introduction

### 1.1. Communication to Improve Patient Safety

Miscommunication is a major patient safety concern. In 2016, The Joint Commission reported communication error as the number one cause of all anesthesia related sentinel events for the period 2004 to 2015 [1]. In the analysis of 444 surgical malpractice claims, 60 cases involved communication breakdown. When multiple healthcare providers across departments and disciplines care for a patient, mistakes in transmitting increasingly complex patient information have been shown to lead to patient harm [2] [3]. Handoff is a transfer of information and professional responsibilities across teams [4]. Despite the wide use of an electronic health record (EHR), a verbal synchronous, face-to-face communication in real-time is a fundamental paradigm of clinical discussion that provides a critical structure and an opportunity for an interactive discussion about a patient [5] [6] [7]. For that reason, The American Society of Anesthesiologist’s standard of care requires the presence of intraoperative anesthesia staff for monitoring during transport and verbal report [8].

From review of surgical malpractice claims, the highest percentage of perioperative mistakes, including 43% of all communication failures, occurs post-operatively as a result of poor handoffs [9]. Studies have shown that poor handoffs increase the risk of patient harm [2] [3] [9] [10] [11] [12] and that standardizing communication protocols for handoffs can decrease medical errors and adverse events [13] [14] [15] [16]. Lack of standard guidelines produces inconsistent reports that are vulnerable to omission of pertinent information. Our objective was to quantify the amount of information transferred. The omission of data has been associated with worse outcomes than passing on poor quality of information [17]. A complete omission of information occurred in 57% of surgical malpractice claims [9]. Observation of handoffs showed items deemed vital components of handoff were reported less than half of the times [18].

Inadequate communication in PACU has been shown to increase morbidity and mortality [19]; a review of 419 reports from Anesthetic Incident Monitoring Study (AIMS) indicated a failure of communication as the second most common contributing factor that led to adverse events in recovery units [10]. PACU is especially vulnerable to communication failures between providers because of physical transfer of patient, collaboration of multiple clinicians, and similar patient histories are common features of PACU [17] [20].

### 1.2. Why a Checklist?

To avoid adverse events caused by miscommunication, The Joint Commission mandated “a standardized approach to handoffs” as a patient safety goal in 2006 [21]. Checklists have been used to standardize consistency in communication among providers and to reduce morbidity and mortality in surgical settings [22]. Checklists in PACU to standardize handoff communications have also been studied to show improvement in data transfer [23] [24] [25] [26] and reduction in medical errors [13] [15] [27]. Multicenter handoff interventions using a handoff checklist in PACU showed decrease in preventable adverse events across disciplines and departments [15] [27]. In addition, use of checklists has shown improvements in nursing staff satisfaction and handoff efficiency [25].

Overall, a checklist accomplishes two goals for both intraoperative and postoperative care providers. First, it provides a guideline that defines a standard for a handoff. Second, a physical checklist is used as a reminder of items to prevent omission of information [18] [26].

### 1.3. Goal & Hypothesis

The goal is to establish measures to decrease perioperative miscommunication and improve patient safety through standardized PACU handoff protocol. We hypothesize that a physical checklist will increase data transfer and efficiency at our PACU, and prevent omission of pertinent patient information in handoff.

## 2. Methods

### 2.1. IRB Approval

IRB exemption was approved by MSKCC under the criteria of observation of public behavior and collection of unidentifiable information of clinician interactions. IRB exemption was approved on May 16, 2016.

### 2.2. Derivation of the Checklist

**Figure 1:**
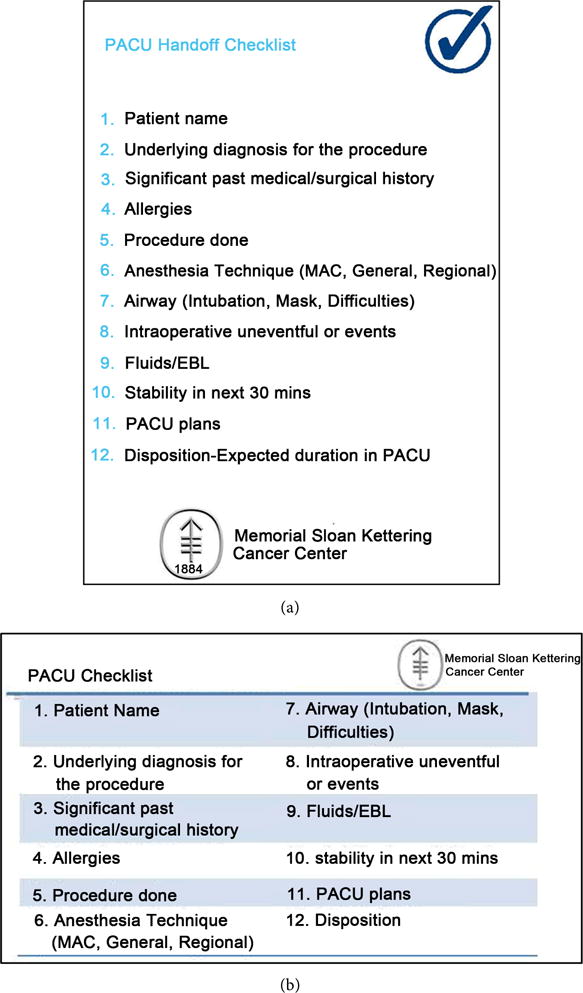
(a) Full-sized checklist that was displayed by every PACU bed; (b) ID badge-sized checklist for post-implementation period.

According to a systemic review of 31 studies on PACU handoff, a handoff should include at minimum: *patient information, anesthesia information, surgical information, current status, and care plan* [17]. A published “Anesthesia Handover Checklist” by Lin and colleagues was used as the initial structural framework. The initial checklist included: *Patient, Underlying diagnosis/procedure*, *Technique-anesthetic*, *Status of procedure*, *Past significant medical history*, *Allergies*, *Timing/expected duration*, *Immediate expected events next* 30 *min*, *Emergence plan*, *Noteworthy aspects of case*, *Treatment plan for post op care*, *Fluids/EBL*, *Induction events*, *Records available for review*, *Signs-vitals*, *and Transfer care to*. These items listed out a mnemonic: PUTS PATIENT FIRST [28]. This checklist included key elements of the transfer of care measures recommended by The American Society of Anesthesiologists [29]. Following the recommendations from various studies that emphasized flexibility in making adjustments according to the implemented institutions [15] [24] [30], the pilot week was used to adjust the working checklist according to the practices at our institution (Figure 1(a)). Multiple iterations were made to test usability and strength of the checklist.

Every item was deemed equally important, and given a score of 1. Although surgical and anesthesia staff received separate grades, the primary endpoint was the total number of checklist items addressed by either department during the PACU handoff. This value ranged from the minimum of 0 to the maximum of 12. For the composite score, if an item is addressed by either a surgical or anesthesia staff, the item is considered to be addressed, and a score of 1 is allocated to the item. For department based scores, the surgical and anesthesia staff reports received separate score of 1 per item accordingly. The start and end time of the handoff was recorded for assessing the duration of handoff rounded to a whole minute. Lastly, every handoff was assigned an unidentifiable number to match the data between two observers.

Total duration of the study included 5 weeks of observation in the main PACU. The first week preceded the study to make adjustments to the checklist as a pilot period. All handovers were observed in real time by two observers. This pilot study yielded 100% consensus on “item qualified” between attending anesthesiologists and the observers. The observers were physically present at all observed handoffs between June 13, 2016 and July 15, 2016 from 10AM to 5PM. Immediately after each handoff, two observers resolved any differences in assessment and arrived at 100% consensus.

### 2.3. Pre-Implementation Period

Second and third weeks served as the control period. Observers gathered data before the checklist was implemented to gauge the current quality of PACU handoff. Both anesthesia and surgical staff being observed were not informed of the reason for the presence of observers in PACU to avoid isolated improvements in handoff behavior.

### 2.4. Post-Implementation Period

After pre-implementation period and prior to the official implementation of the checklist, all anesthesia staff–including attending anesthesiologist, Certified Registered Nurse Anesthetists (CRNAs), resident physicians, and CRNA students-were made aware of the study. They were provided an electronic and physical copy of the checklist. An A4-sized laminated checklist was available by every patient bed. Additionally, ID badge-sized checklists were distributed to every provider (Figure 1(b)). During the last two weeks of the study, anesthesia staff were asked to use the checklist by the observers before each report. Surgical staff were not part of the intervention although data were collected from their reports.

### 2.5. Statistical Analysis

A traditional randomized study would randomize each handoff 1:1 to the group with or without a checklist. However, this approach would require some staff to unlearn the checklist hints after being exposed to it. Because of these logistical and feasibility issues, our study is based on convenience sampling in that the first two weeks of the study was pre-implementation of the checklist, while the second two weeks was post-implementation. This approach assumes that the case mix (patient and surgical characteristics) is similar between the two phases of the study, which is reasonable in this high volume cancer center. Power calculation was performed prior to the study to determine the minimal detectable difference (MDD) necessary to achieve 80% and 90% power for a two-sided t-test, given 50 patients in each arm and type I error rate of 0.05. The MDD refers to the smallest treatment effect that can be identified assuming a known sample size. We assume mean of 5 items completed in the pre-implementation phase, with a conservative standard deviation (SD) of 5. The MDD are 2.85 and 3.25 for 80% and 90% power. This translates to an assumed mean of 7.85 and 8.25 checklist items completed in the post-implementation phase. If instead SD is 2, the MDD changes to 1.14 and 1.30 for 80% and 90% power.

We examined each item individually to identify the proportion of handoffs addressed for the specific item between the two groups using Fisher’s exact test. The secondary outcome of duration of PACU handoff is compared between the two groups using Wilcoxon rank sum test. As exploratory analyses, we compare the total number of items addressed in the post-implementation phase by consistency status to assess whether consistency impacts the quality of handoff. All analyses were repeated with the component scores which included the items addressed by the anesthesia provider only. All statistical tests were two-sided at alpha level of 0.05, performed using Stata 13 (Stata Corp, College Station, TX).

## 3. Results

### 3.1. Data Transfer

**Figure 2:**
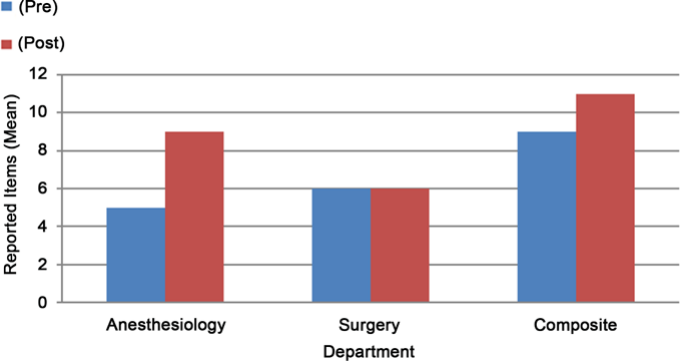
Post-implementation of the checklist, median reported items increased from nine to eleven in composite values. Abbreviation: Pre: pre-implementation; Post: post-implementation.

We observed a total of 120 PACU handoffs. 60 handoffs were each observed pre-implementation and post-implementation of the checklist. Composite values analyzed items as addressed by either surgical or anesthesia staff. Department based values analyzed items addressed by surgical and anesthesia staff separately. Pre-implementation of a checklist, the composite value showed a mean of 8.7 (SD = 1.5) items reported out of a total of 12 items on the checklist, and post-implementation the median report increased to 10.9 items (Table 1) (Figure 2). When anesthesia staff reports were analyzed independently, the mean reported items increased from 4.8 (SD = 1.6) to 8.9 (SD = 2.0) items post-implementation of the checklist (Table 1). Interestingly, surgical staff report stayed relatively consistent at mean of 5.9 items pre and 5.5 items post-implementation periods. In the analysis of anesthesia staff reports, items that were consistently reported at low numbers despite the checklist were: PACU Plans, Disposition-Expected Duration in PACU, Underlying Diagnosis, and Procedure Done (Table 2).

From the composite values, most improvements were seen with the following items: Allergies, Anesthesia Technique, and Airway (Table 2). In pre-implementation composite values, Allergies, Anesthesia Technique, and Airway were reported during 63% (38, N = 60), 58% (35, N = 60), and 57% (34, N = 60) of the handoffs respectively. Post-implementation of a checklist, reports about Allergies, Anesthesia Technique, and Airway all increased to 93% (56, N = 60, *p* <0.0001). In contrast, the least improvements in composite value were noted with Patient Name and Stability in 30 Minutes. Pre-implementation of a checklist, Patient Name and Stability in 30 Minutes were mentioned 55% (33, N = 60) and 20% (12, N = 60) of the handoffs, respectively. Post-implementation of a checklist, Patient Name and Stability in 30 Minutes increased to 88% (53, N = 60, *p* < 0.0001) and 68% (41, N = 60, *p* < 0.0001) respectively (Table 2).

### 3.2. Secondary Analysis

Duration of each handoff is the total time of both surgical and anesthesia staff reports rounded to a minute. Post-implementation, the median duration of handoff, is increased by one minute (Table 3). However, in comparison to the increase in the number of reported items with longer handoffs during the pre-implementation period, duration of the handoffs was independent of the number of reported items post-implementation (Table 4).

## 4. Discussion

Our results demonstrate that, in the setting studied, the use of a checklist improved the overall quantity of data transfer during PACU handoff. A checklist was introduced and implemented for two weeks. During the post-implementation period, more items were reported in all intervals of “handoff duration” in comparison to pre-implementation period. Using a checklist to prevent omission of patient information during handoff is important because miscommunication from multiple care transfer has been shown to increase patient harm [2] [3] [9] [10] [11] [12]. To mitigate these adverse events, a use of checklist in PACU has shown not only an increase in data transfer [23] [24] [25] [26] but also a decrease in medical errors [13] [14] [15]. Our study adds that a checklist reminds the staff of defined standard of items to report in order to minimize information omission during PACU handoff. Furthermore, the quantity of minimum data transferred during handoff is independent of the quality of the reports, which is more closely associated with complexity of patient history and not an objective of this study.

### 4.1. Quantity of Data Transfer

There are two important reasons for analyzing the data as composite values and department based values. First, PACU handoff is provided by both the surgical and anesthesia team at our institution. The median number of items reported by surgical staff stayed the same at a median of 6 items out of the total 12 during post-implementation period. This data indicates that the increase in overall improvement in handoff during post-implementation can be attributed to improvements in anesthesia reports without unintended observational influence on the quality of surgical staff reports. Quality of anesthesia reports as a standalone report is also important because collaborative report may not be a standard of practice at all institutions. In many institutions, only anesthesia gives report during PACU handoff. As an ideal standard, anesthesia staff should be able to adequately report surgical information in case anesthesia staff is the only informant of intraoperative events.

**Figure 3:**
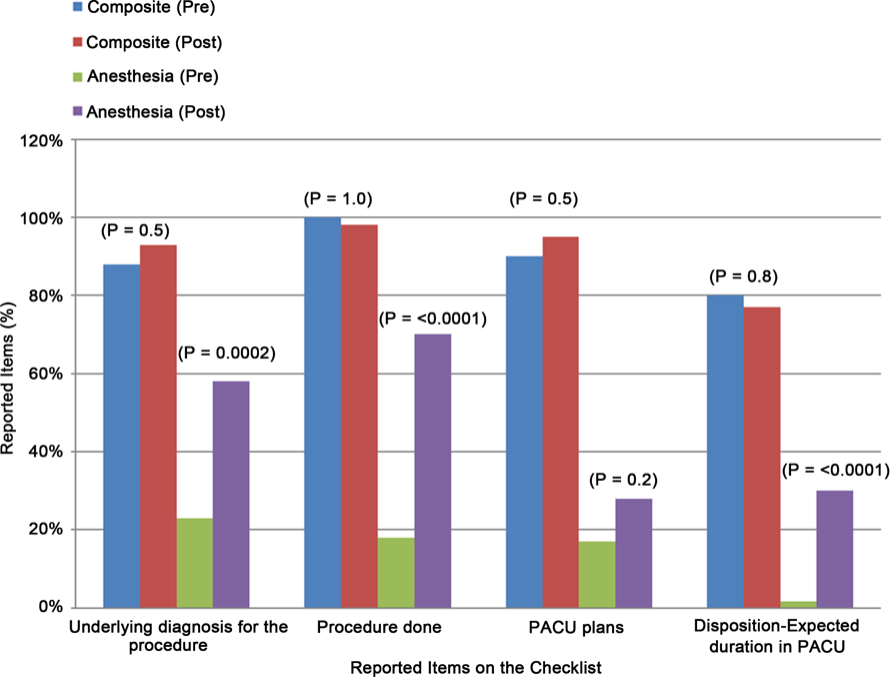
Analysis of the anesthesia reports showed least improvements with items related to surgical practice. Pre: pre-implementation; Post: post-implementation.

**Figure 4:**
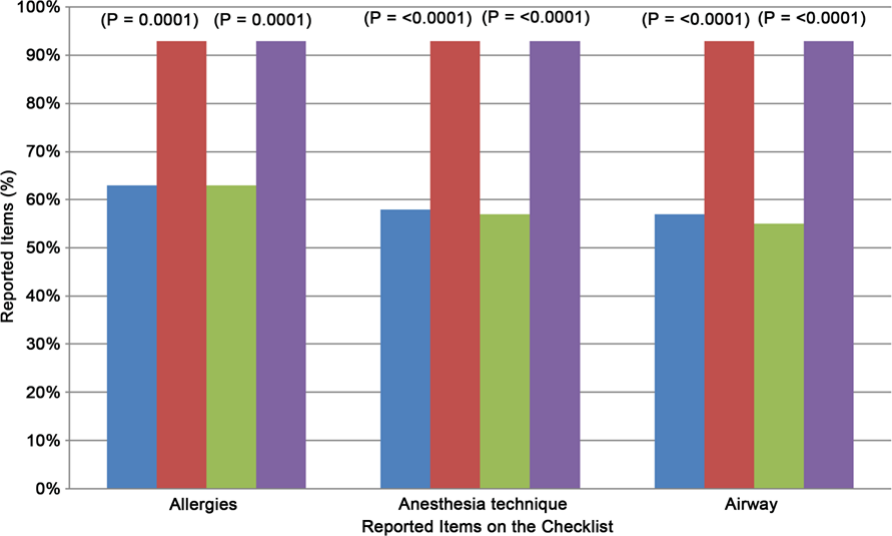
Analysis of the anesthesia reports showed most improvements with items related to anesthesia practice. Pre: pre-implementation; Post: post-implementation.

Second, comparing composite and department based values helped us to identify items pertinent to either surgical or anesthesia staff during handoffs. Items related to surgical procedure improved the least in the anesthesia staff reports (*i.e.* PACU plans, Disposition-Expected Duration in PACU, Underlying diagnosis, and Procedure done) (Figure 3). Because compliance of reporting surgical information by anesthesia staff was lowest in a similar study, the data was used to recommend presence of surgical staff during handoffs [31]. Items relevant to the practice of anesthesia improved the most in anesthesia reports and composite value (*i.e.* Allergies, Anesthesia Technique, and Airway) (Figure 4). Considering that prior to implementation of a checklist, about half of the reports did not include these anesthesia specific information, a checklist reduced omission of at least the most relevant information.

### 4.2. Duration

Contradictory to our hypothesis that a checklist would reduce the duration of a handoff, we observed an overall increase in median time spent during a handoff. Previous studies have shown conflicting reports on the effect of handoff duration after implementing a checklist [23] [25] [26] [31].

The lack of training in using the checklist led to providers stumbling or pausing during the report. Majority of the informal feedback from the anesthesia staff were disturbances to their original “flow” with a different order of items and some unfamiliar items on the checklist. Despite the foreseen improvements in handoff with a physical checklist [26], multimodal staff training models could improve incorporating a new checklist to practice [24] [25] [27] [32]. Second, we recommend multiple Plan-Do-Study-Act cycles24 and incorporating staff feedback18 to design a checklist that better fits the context of each institution.

### 4.3. Limitations

Hawthorne effect is the influence of the presence of an observer on observed behavior. During pre-implementation phase, anesthesia staff was given limited details on what we were observing. We cannot overlook the influence of the presence of the two observers during all observed handoffs. It is likely that data transfer improved for our control group simply because of Hawthorne effect. For instance, we can assume that the observed handoffs were more comprehensive than the baseline handoffs before our study. It follows then, that the actual improvement in handoff is indeed better than we reported. We expect to find more pronounced improvement if our results were subject to the Hawthorne effect.

Although prior studies have correlated adverse events with poor handoff [2] [3], direct comparison to patient outcome with a handoff checklist in a randomized trial is ideal but difficult to accomplish.33 Thus, deriving from previous studies, we assume that our improvement in data transfer will decrease adverse events and improve patient outcome. Secondly, although complexity of the patient history may take longer to report, patient population was not defined or restricted [33]. Patients may vary greatly in their phase of cancer treatment at our institution. Our data is based on the local context and exact results cannot be generalized for other institutions without further multicenter investigation [34]. Thirdly, creating definitive categories of medications or tools with the help of practicing anesthesiologists, observers were taught to convert qualitative data from observations to quantitative data during the pilot week. Despite the 100% consensus made between two observers after every handoff, we cannot neglect the human variables in information gathering.

## 5. Conclusion

In conclusion, implementation of a physical checklist for PACU handoff increased overall data transfer and prevented omission of patient information. Report duration did not have an impact on overall data transfer. For future directions, we recommend incorporating staff feedback into Plan-Do-Study-Act cycles for an improved checklist to ensure compliance and familiarize the staff with the use of a checklist through multimodal training modules.

## Figures and Tables

**Table 1:** Summary of median and 25^th^, 75^th^ percentiles of reported items^a^.

	Composite Value			Anesthesia Reports Only			Surgery Reports Only		
	Pre^b^	Post	P Value	Pre	Post	P Value	Pre	Post	*P* Value
Mean (SD)	8.7 (1.5)	10.9 (1.1)	<0.0001	4.8 (1.6)	8.9 (2.0)	<0.0001	5.9 (1.6)	5.5 (1.7)	0.2
25^th^, 75^th^ percentiles	8.0, 10.0	10.0, 12.0		4.0, 6.0	7.5, 10.0		5.0, 7.0	4.0, 7.0	
Median	9.0	11.0	<0.0001	5.0	9.0	<0.0001	6.0	6.0	0.4
<12 items	59 (98%)	39 (65%)	<0.0001	60 (100%)	51 (85%)	0.003	60 (100%)	60 (100%)	NA
12 items	1 (1.7%)	21 (35%)		0 (0%)	9 (15%)				
<11 items	53 (88%)	20 (33%)	<0.0001	60 (100%)	47 (78%)	0.0001	60 (100%)	60 (100%)	NA
11 items or <	7 (12%)	40 (67%)		0 (0%)	13 (22%)				
<10 items	43 (72%)	6 (10%)	<0.0001	59 (98%)	38 (63%)	<0.0001	60 (100%)	60 (100%)	NA
10 items or <	17 (28%)	54 (90%)		1 (1.7%)	22 (37%)				

The reported items of composite value increased from a mean of 8.7 items to 10.9 items post-implementation of the checklist. Minimum number of reported items increased in the composite value and anesthesia reports post-implementation of the checklist. Pre: pre-implementation; Post: post-implementation.

a N = 12 items;

b N = 60 handoffs in every pre and post categories.

**Table 2:** Breakdown of handoffs by reported items.

Items	Composite Value			Anesthesia Reports Only			Surgery Reports Only		
	Pre	Post	*P* Value	Pre	Post	P Value	Pre	Post	*P* Value
1. Patient Name	33 (55%)	53 (88%)	<0.0001	14 (23%)	51 (85%)	<0.0001	29 (48%)	23 (38%)	0.4
2. Underlying diagnosis for the procedure	53 (88%)	56 (93%)	0.5	14 (23%)	35 (58%)	0.0002	51 (85%)	47 (78%)	0.5
3. Significant past medical/surgical history	51 (85%)	59 (98%)	0.017	41 (68%)	55 (92%)	0.002	38 (63%)	34 (57%)	0.6
4. Allergies	38 (63%)	56 (93%)	0.0001	38 (63%)	56 (93%)	0.0001	2 (3.3%)	6 (10%)	0.3
5. Procedure done	60 (100%)	59 (98%)	1	11 (18%)	42 (70%)	<0.0001	60 (100%)	57 (95%)	0.2
6. Anesthesia technique	35 (58%)	56 (93%)	<0.0001	34 (57%)	56 (93%)	<0.0001	8 (13%)	2 (3.3%)	0.095
7. Airway	34 (57%)	56 (93%)	<0.0001	33 (55%)	56 (93%)	<0.0001	1 (1.7%)	0 (0%)	1
8. Intraoperative uneventful or events	44 (73%)	54 (90%)	0.032	30 (50%)	49 (82%)	0.0005	31 (52%)	34 (57%)	0.7
9. Fluids/EBL	59 (98%)	60 (100%)	1	59 (98%)	60 (100%)	1	22 (37%)	20 (33%)	0.8
10. Stability in next 30 mins	12 (20%)	41 (68%)	<0.0001	5 (8.3%)	36 (60%)	<0.0001	9 (15%)	6 (10%)	0.6
11. PACU plans	54 (90%)	57 (95%)	0.5	10 (17%)	17 (28%)	0.2	53 (88%)	56 (93%)	0.5
12. Disposition-Expected duration in PACU	48 (80%)	46 (77%)	0.8	1 (1.7%)	18 (30%)	<0.0001	48 (80%)	44 (73%)	0.5

Most improved reported items in composite value were items related to anesthesia, such as: patient name, allergies, anesthesia technique, and airway. Pre: pre-implementation; Post: post-implementation.

**Table 3:** Summary of Handoff Duration (minute)^a^.

	N	Mean	SD	Median	Min	Max
Pre	60	2.9	1.3	3	1	7
Post	60	3.9	1.8	4	1	9

Overall duration of a handoff increased during the post-implementation period of the checklist. Pre: pre-implementation; Post: post-implementation; SD: standard deviation; Min: minimum; Max: maximum.

a Duration included both surgical and anesthesia reports.

**Table 4:** Breakdown of handoff duration^a^^,^^b^.

	Pre			Post		
	
Duration	Median	25^th^, 75^th^ percentile	Percentage	Median	25^th^, 75^th^ percentile	Percentage
1 min	6.5	6.0, 9.0	(N = 8; 73%)	11.0	10.0, 12.0	(N = 3; 27%)
2 min	8.5	7.5, 9.0	(N = 16; 62%)	12.0	10.0, 12.0	(N = 10; 38%)
3 min	9.0	8.0, 10.0	(N = 21; 58%)	11.0	10.0, 11.0	(N = 15; 42%)
4 min	9.5	9.0, 10.0	(N = 8; 36%)	11.0	11.0, 12.0	(N = 14; 64%)
5 min	8.0	8.0, 9.0	(N = 5; 45%)	11.0	10.0, 12.0	(N = 6; 55%)
6 min	10.0	10.0, 10.0	(N = 1; 13%)	11.0	10.0, 12.0	(N = 7; 88%)
7 min	11.0	11.0, 11.0	(N = 1; 25%)	11.0	10.0, 11.0	(N = 3; 75%)
8 min	–			–		
9 min	–			11.5	11.0, 12.0	(N = 2; 100%)

Improvements in handoff during the post-implementation period were independent of the duration. Pre: pre-implementation; Post: post-implementation.

a Duration included both surgical and anesthesia reports;

b N = 12.

## Abbreviation List

PACU: Post Anesthesia Care Unit
MSKCC: Memorial Sloan Kettering Cancer Center
EHR: Electronic Health Record
AIMS: Anesthetic Incident Monitoring Study
IRB: Institutional Review Board
CRNA: Certified Registered Nurse Anesthetist
MDD: Minimal Detectable Difference
SD: Standard Deviation
